# Recurrent Embolic Strokes in Mechanical Aortic Valve: Diagnostic and Management Challenges

**DOI:** 10.1016/j.jaccas.2025.105829

**Published:** 2025-10-25

**Authors:** Pernille G. Eriksen, Dorte Damgaard, Mariann Tang, Jonas A. Povlsen, Jesper Møller, Bjarne L. Nørgaard, Ivy S. Modrau

**Affiliations:** a Department of Clinical Medicine, Cardiothoracic and Vascular Surgery, Aarhus University, Aarhus, Denmark; b Department of Neurology, Aarhus University Hospital, Aarhus, Denmark; c Department of Cardiothoracic and Vascular Surgery, Aarhus University Hospital, Aarhus, Denmark; d Department of Cardiology, Aarhus University Hospital, Aarhus, Denmark

**Keywords:** anticoagulation, aortic valve, stroke, thrombosis, valve replacement

## Abstract

**Background:**

Recurrent embolic strokes without an identifiable source in patients with mechanical heart valves pose a significant diagnostic challenge.

**Case Summary:**

A 59-year-old woman with a mechanical aortic valve experienced recurrent embolic strokes, the first nearly 7 years after implantation during subtherapeutic anticoagulation. Repeated neurologic and cardiac imaging, thrombophilia screening, and prolonged rhythm monitoring failed to identify a definitive embolic source. Fluoroscopy and computed tomography showed discrete leaflet opening restriction. Multifocal infarctions raised clinical suspicion for mechanical valve thrombosis, prompting surgical exploration that confirmed diagnosis. The valve was replaced with a bioprosthesis, and left atrial appendage occlusion was performed. The patient remained event-free at 8-month follow-up.

**Discussion:**

Mechanical valve thrombosis is rare and often undetectable. This case highlights the diagnostic dilemma and importance of multidisciplinary team evaluation.

**Take-Home Message:**

Recurrent embolic strokes in mechanical valve patients with inconclusive testing should prompt multidisciplinary evaluation and consideration of valve replacement.

## Visual Summary

**Figure:**
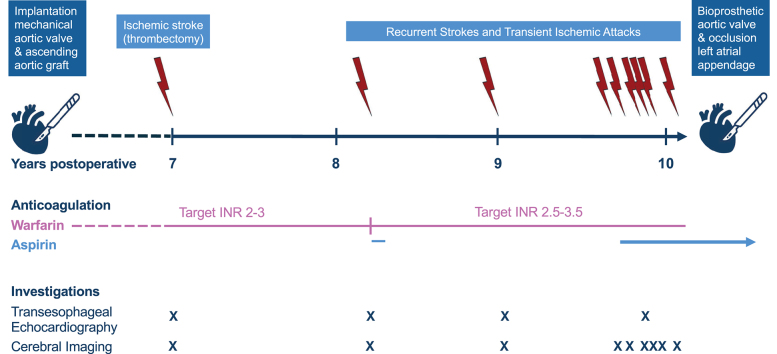

## History of Presentation

A 59-year-old woman with a mechanical aortic valve presented with a series of recurrent cerebral thromboembolic events beginning nearly 7 years after valve implantation. The initial stroke occurred in the setting of subtherapeutic anticoagulation (international normalized ratio [INR]: 1.4) and resolved completely after successful recanalization with thrombectomy. Over the next 3 years, the patient experienced further 8 cerebral thromboembolic events despite sustained—and later intensified—anticoagulation treatment. Vital signs, including blood pressure, remained stable throughout. After a cluster of recurrent events, redo valve replacement was pursued based on clinical suspicion of mechanical valve thrombosis, despite a lack of definitive imaging evidence.

**Take-Home Messages**

• Current noninvasive cardiac imaging often fails to detect small, nonobstructive thrombi on mechanical aortic valves.
• Recurrent embolic events despite negative imaging warrant individualized evaluation by a multidisciplinary team and consideration of surgical intervention.

## Past Medical History

Ten years before presentation, the patient underwent implantation of a 22-mm bileaflet mechanical aortic valve (ATS Open Pivot AP, advancing the standard; ATS Medical Inc) and a 26-mm ascending aortic tube graft for stenotic bicuspid valve with ascending aortic dilation. Cardiovascular risk factors included medically treated hypertension and hypercholesterolemia. She developed intermittent left bundle branch block, which later became persistent. Cardiac computed tomography excluded coronary artery disease; left ventricular ejection fraction remained preserved. Other comorbidities included nonpharmacologically managed intermittent depression, psoriatic arthritis treated with methotrexate, chronic back pain, and prior lumbar discectomy.

**Figure 4:**
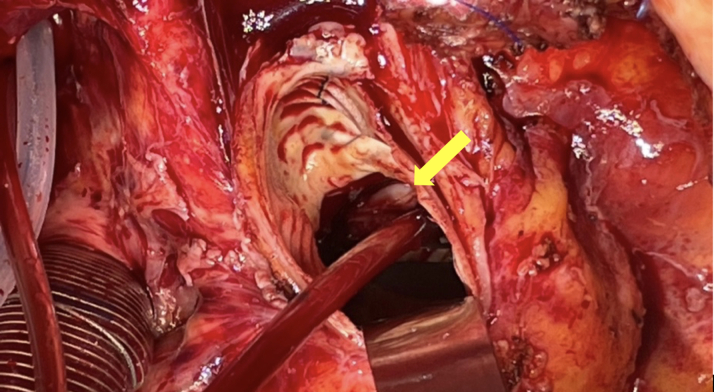
Thrombotic Material on Aortic Annulus After Valve Explantation Intraoperative image of the aortic annulus after explantation of the mechanical aortic valve, revealing consolidated thrombotic material involving approximately one-third of the annular circumference.

## Differential Diagnosis

Key differential diagnoses include mechanical valve thrombosis, atrial fibrillation, infective endocarditis, thrombophilia and other hypercoagulable states, paradoxical embolism, aortic dissection, mural thrombus, large-vessel atherosclerosis, and cryptogenic stroke.

## Investigations

**Figure 1:**
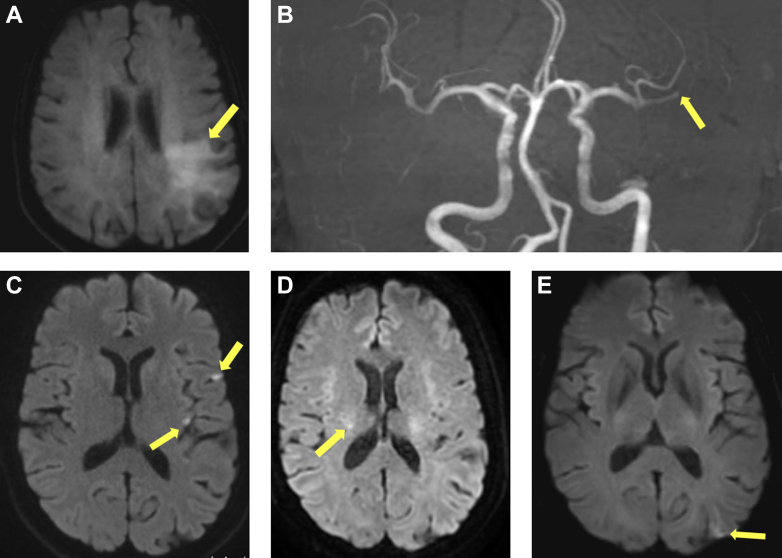
Brain Magnetic Resonance Imaging demonstrating Recurrent Multifocal Ischemic Events Brain magnetic resonance imaging (MRI) demonstrating multifocal ischemic events during the clinical course. A and B correspond to the initial stroke; C to E represent selected imaging from transient ischemic attack 5, 7, and 8. (A) Diffusion-weighted MRI showing acute ischemia in the left insular and parietal cortex. (B) MRI (time-of-flight angiography) demonstrating thrombus in the left M2 segment. (C) MRI revealing 2 of 3 silent cortical infarcts in the left hemisphere. (D) MRI showing punctate diffusion restriction in the right corona radiata. (E) MRI showing cortical diffusion restriction in the left hemisphere. M2 = 2nd segment of the middle cerebral artery.

**Figure 2:**
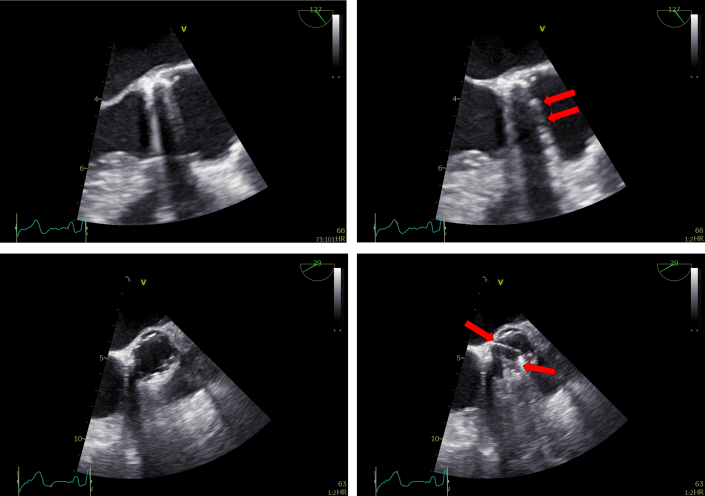
Transesophageal Echocardiography High-esophageal long-axis views (upper panels) and short-axis views (lower panels) are displayed in diastole (left panels) and systole (right panels). The red arrows highlight symmetric disk motion. Leaflet mobility is preserved, with no paravalvular leak or other evidence of prosthetic valve dysfunction.

**Figure 3:**
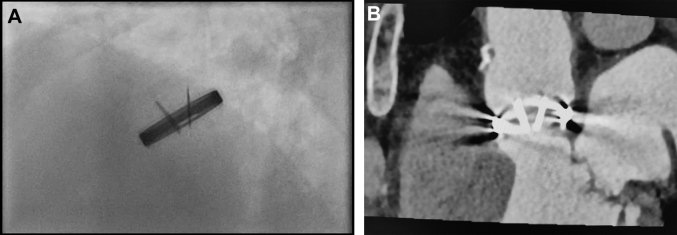
Fluoroscopic and CT Evaluation of the Implanted Bileaflet Mechanical Valve Fluoroscopic (A) and multidetector computed tomography (B) assessment of the implanted bileaflet mechanical valve. Both imaging modalities revealed reduced leaflet opening, with one leaflet at 60° to 65° and the other at 70° to 75°. According to the manufacturer, the leaflets open to 85° when fully functional, whereas normally functioning ATS valves in vivo open to approximately 70°,^10^ highlighting the subtle restriction of the first leaflet.

Repeated brain magnetic resonance imaging revealed multifocal ischemic lesions (Table 1, Figure 1). Serial transthoracic echocardiography (TTE) and transesophageal echocardiography (TEE) (Video 1), as well as electrocardiogram (ECG)-gated multidetector cardiac computed tomography (MDCT), showed no evidence of prosthetic obstruction, pannus, or thrombus (Figure 2). MDCT was performed with a dual source computed tomography scanner (Siemens Somatom Force) using an ECG-gated full-cycle retrospective protocol and high tube voltage (140 kV) to counteract valve artifacts. However, cinefluoroscopy (Video 2) and MDCT (Video 3) were consistent in showing a 10° reduction in opening angle of one leaflet, raising concern for subtle mechanical restriction (Figure 3). Repeated ECGs and extended Holter monitoring confirmed sustained sinus rhythm. Duplex ultrasound and computed tomography angiography showed no evidence of carotid artery disease. Thrombophilia and coagulation testing were unremarkable. Despite this comprehensive work-up, the embolic source remained undetermined until mechanical valve thrombosis was confirmed intraoperatively.

**Table 1:** Clinical Timeline and Diagnostic-Therapeutic Course

Event No.	Time Point	Clinical Event	Presenting Symptoms	Diagnostic Work-Up			Intervention
				Cardiac	Cerebral	Other	
	Baseline	Mechanical aortic valve replacement (22 mm) and ascending aortic tube graft (26 mm)					Lifelong warfarin—target INR 2-3 Self-management training
1	+6 y, 9 mo	Ischemic stroke INR: 1.4	Aphasia, right-sided hemisensory deficits	TEE: Unchanged No signs of valve thrombosis	Brain MRI: 1. Thrombus in a left M2 segment 2. Diffusion restriction in left insular and parietal cortex 24-hour brain MRI: 3 small DWI lesions in the same region	ECG: SR, LBBB	Thrombectomy (recanalization) LMWH bridging Warfarin continued—target INR 2-3 Stroke rehabilitation
2	+8 y, 3 mo	Clinical suspicion of TIA	Transient paresthesia left arm followed by right-sided headache	TEE: Unchanged No signs of valve thrombosis	Brain MRI: No diffusion restrictions Transcranial Doppler: Microembolism in right intracranial arteries Carotid US: Minor plaque in the proximal left ICA	ECG: SR, narrow QRS complexes	Suppl. aspirin for 1 month (75 mg daily) Warfarin—target INR 2.5-3.5
3	+9 y	Clinical suspicion of TIA	Paresthesia left arm	TTE: Normal aorta valve prosthesis TEE: Unchanged. No signs of valve thrombosis Myocardial perfusion imaging: Normal	Brain MRI: Silent punctate diffusion restriction in the left medial temporal lobe MRA cervical and intracerebral arteries: Normal Transcranial Doppler: Microembolism in right intracranial arteries	ECG: SR, narrow QRS Holter monitoring: SR, rare PAC and PVC	Warfarin continued
4	+9 y, 8 mo	Clinical suspicion of TIA	Transient right leg motor impairment with foot drop		Brain CT: No acute findings Carotid US: No significant carotid stenosis	ECG: SR, LBBB	Warfarin continued
5	+9 y, 10 mo	Clinical suspicion of TIA	Acute-onset headache, transient weakness, and paresthesia right leg		Brain CT: Normal	ECG: SR, LBBB	Warfarin continued
6	+9 y, 11 mo	Ischemic stroke	Acute-onset frontal headache with paresthesia right leg		Brain CT: No acute findings Brain MRI: 3 silent cortical diffusion restrictions in the left hemisphere	ECG: SR, LBBB	
7	+9 y, 11 mo	Ischemic stroke	24-hour before admission: transient right visual field defect At admission: transient left upper extremity weakness, left-sided paresthesia, dysarthria, and lip tingling	TTE: Normal aortic valve prosthesis TEE: Unchanged. No signs of valve thrombosis Multidetector cardiac CT and fluoroscopy: Both discs mobile but 10° angle difference	Brain MRI: 2 punctate diffusion restrictions in left occipital and frontal regions. Microbleed in right thalamus and left frontal region CT angiography of carotid and intracerebral arteries: Normal	ECG: SR, LBBB Holter monitoring: SR, rare PVC Thrombophilia work-up deferred—no impact on management	Warfarin continued Suppl. low-dose aspirin for 1 month
	+9 y, 11 mo	Multidisciplinary Team Conference					
8	+9 y, 11 mo	Ischemic stroke	Left-sided hemiparesis and facial paresis, dysarthria		Brain MRI: Punctate diffusion restriction in right corona radiata	ECG: SR and LBBB Thrombophilia work-up: Normal	Warfarin continued Aspirin continued for 3 months
	+10 y	Second opinion at a University Hospital, Department of Neurology					
9	+10 y, 1 mo	Ischemic stroke	Right homonymous hemianopia		Brain MRI: Cortical diffusion restriction in left hemisphere		Warfarin and aspirin continued
	+10 y, 2 mo	Bioprosthetic aortic valve (21 mm) and prophylactic occlusion left atrial appendage					Cardiac Rehabilitation Lifelong Aspirin therapy

CT = computed tomography; DWI = diffusion-weighted imaging; ECG = electrocardiogram; ICA = internal carotid artery; INR = international normalized ratio; LBBB = left bundle branch block; LMWH = low molecular weight heparin; M2 = 2nd segment of the middle cerebral artery; MRA = magnetic resonance angiography; MRI = magnetic resonance imaging; PAC = premature atrial contraction; PVC = premature ventricular contraction; SR = sinus rhythm; TEE = transesophageal echocardiogram; TIA = transient ischemic attack; TTE = transthoracic echocardiogram; US = ultrasound.

**Visual Summary:**
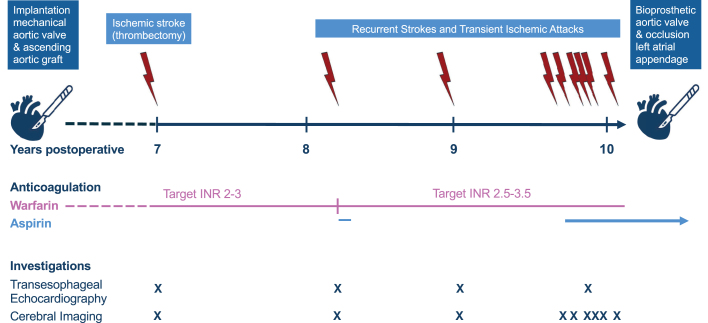
Timeline of Events Timeline of clinical events, anticoagulation adjustments, and diagnostics after mechanical valve implantation, ending with redo valve replacement and left atrial appendage occlusion. Arrows = key events; x = diagnostic test.

## Management

After mechanical valve replacement, the patient initiated lifelong warfarin with self-managed anticoagulation targeting an INR of 2.0 to 3.0. Approximately 1 year before the initial stroke, depressive symptoms led to inconsistent INR monitoring and subtherapeutic control despite a continued fixed-dose regimen.

After this stroke, she received re-education in self-management. Recurrent cerebral thromboembolic events occurred despite therapeutic anticoagulation, prompting escalation of the INR target to 2.5 to 3.5 and addition of low-dose aspirin, in line with current guidelines.^1^

Multifocal ischemic lesions across distinct vascular territories suggestive of a cardioembolic source prompted the multidisciplinary team to recommend redo surgery. The decision followed a shared decision-making process with active patient involvement based on the clinical suspicion of mechanical valve thrombosis despite inconclusive evidence.

Surgical re-exploration involved replacement of the mechanical valve with a bioprosthetic valve and prophylactic left atrial appendage occlusion. Intraoperative inspection showed normal valve function and a subvalvular thrombus involving approximately one-third of the annular circumference (Figure 4), confirmed by histopathology. Postoperatively, warfarin was discontinued and low-dose aspirin monotherapy maintained.

## Outcome and Follow-Up

Recovery was uneventful except for complete heart block requiring permanent pacemaker implantation 14 days postoperatively. TTE demonstrated a well-functioning bioprosthetic aortic valve, preexisting mild mitral regurgitation, and preserved ventricular function.

Eight months postoperatively, the patient had completed cardiac rehabilitation and remained free of thromboembolic events. Aspirin monotherapy was maintained.

## Discussion

This case illustrates the complexity of diagnosing mechanical valve thrombosis and managing recurrent cerebral thromboembolic events in patients with mechanical aortic valves. Although the initial ischemic stroke occurred during a period of subtherapeutic anticoagulation, it was followed by multiple thromboembolic events despite sustained therapeutic INR and escalation of antithrombotic treatment.

Repeated cardiac imaging failed to reveal definitive thrombus despite increasing clinical suspicion of cardioembolic events. Diagnosis was ultimately confirmed intraoperatively, underscoring limitations of current imaging modalities.

Mechanical valve thrombosis is an uncommon but potentially life-threatening cause of thromboembolism and acute valve dysfunction, with thromboembolic events occurring in approximately 5% of patients with mechanical aortic valves, with the highest risk occurring in the early postoperative period.^2^ Limitations of current imaging modalities remain a central challenge in the timely diagnosis and management of mechanical valve thrombosis. Although TEE remains the gold standard for prosthetic valve assessment, it often fails to directly visualize thrombus. Diagnosis frequently relies on indirect findings, including restricted leaflet motion, abnormal transvalvular flow pattern, elevated gradients, or central regurgitation.^3^ Sensitivity is particularly limited for detecting small, nonobstructive thrombi in mechanical aortic valve prostheses, with reported miss rates of 17% to 50% and up to 80% for TTE.^3^^,^^4^ Technical factors such as acoustic shadowing, suboptimal leaflet visualization, and difficulty distinguishing thrombus from pannus or imaging artifacts contribute to limitations.^4^

In the present case, repeated TTE, TEE, and supplemental MDCT failed to identify the thrombus despite increasing clinical suspicion of a cardiac embolic source. This underscores the limited sensitivity of current cardiac imaging modalities due to spatial resolution constraints and imaging artifacts.^4^, ^5^, ^6^ The present subvalvular thrombus was circumferential and closely adherent to the annular wall, which likely contributed to its being overlooked. Retrospectively, the subtle reduction in one leaflet's opening observed on fluoroscopy and MDCT—though not confirmed intraoperatively—may have warranted greater diagnostic consideration.^3^^,^^6^

The therapeutic implications of these diagnostic challenges are significant. Current European guidelines recommend redo surgery primarily for obstructive mechanical valve thrombosis or embolism associated with thrombi larger than 10 mm but offer limited guidance for nonobstructive thrombi or cumulative embolic burden.^1^ This gap may prolong exposure to thromboembolic risk due to diagnostic uncertainty and hesitancy to deviate from established treatment algorithms. Recurrent strokes in this patient resulted in multiple acute hospital admissions and substantial distress and anxiety, reflecting an existential threat despite the absence of persistent neurologic deficits. These limitations highlight the need for a more nuanced, risk-adapted approach beyond existing guideline thresholds. Clinicians should recognize these diagnostic limitations, maintain a high index of suspicion, and engage a multidisciplinary team involving cardiology, neurology, and cardiac surgery with coagulation experts consulted as needed. Shared decision-making with the patient should incorporate embolic history, individualized risk-benefit analysis, and quality-of-life considerations to inform patient-centered management.

The initial ischemic stroke occurred during a period of depressive mood that compromised the patient's otherwise well-managed self-anticoagulation, resulting in subtherapeutic INR. Although self-management of warfarin has been associated with improved time in therapeutic range and reduced thromboembolic complications in selected patients, its effectiveness relies on adherence and psychological stability. In patients with mechanical heart valves, nonadherence remains a major contributor to adverse events, with depression and insufficient knowledge about anticoagulation identified as key risk factors.^7^ Consequently, it has been proposed that routine evaluation of both adherence and mental health should be integrated into the follow-up of patients on long-term warfarin therapy to ensure safe and effective anticoagulation.^7^

Reluctance to pursue redo aortic valve replacement may reflect overestimation of the surgical risk. However, excluding infective endocarditis, contemporary data show excellent outcomes, with operative mortality as low as 2.5% and pacemaker implantation rates below 4%.^8^ In series reporting higher mortality, the primary determinants of adverse outcomes were endocarditis and operative urgency.^9^ This supports earlier surgical consideration in patients with recurrent embolic events and acceptable surgical risk, particularly when medical therapy has been exhausted.

## Conclusions

Nonobstructive thrombosis of mechanical aortic valves remains a diagnostic challenge due to the limited sensitivity of current imaging modalities. In cases of recurrent embolic events with inconclusive work-up, sustained clinical suspicion of a cardiac source should prompt multidisciplinary team evaluation with active patient involvement. Shared decision-making may support surgical re-exploration to secure a definitive diagnosis and prevent further embolic complications.

## Funding Support and Author Disclosures

The authors have reported that they have no relationships relevant to the contents of this paper to disclose.

## Appendix

Video 1 Transesophageal Echocardiography of the Mechanical Valve Short-axis view demonstrating symmetric disk motion, preserved leaflet mobility, and no paravalvular leak or other evidence of prosthetic valve dysfunction.

Video 2 Cinefluoroscopic Assessment of the Mechanical Aortic Valve Long-axis projection demonstrates dynamic opening and closing of both leaflets, with one showing a subtle 10° reduction in opening angle compared with the other.

Video 3 Multidetector Computed Tomography Assessment of Mechanical Valve Function Dynamic assessment of the bileaflet mechanical valve on computed tomography showed restricted motion, with one leaflet showing a subtle 10° reduction in opening angle compared with the other.
